# Cultural immersion and AI-supported instruction: international students’ Chinese as a foreign language enjoyment

**DOI:** 10.3389/fpsyg.2026.1814709

**Published:** 2026-10-02

**Authors:** Junfeng Jiao, Meilan Liu

**Affiliations:** 1 School of Foreign Languages, Jianghan University, Wuhan, China; 2 School of Humanities and Foreign Languages, China Jiliang University, Hangzhou, China

**Keywords:** Chinese as a foreign language enjoyment (CFLE), cultural immersion and AI-supported instruction, emotion-motivation, international students, SDT

## Abstract

**Introduction:**

While Foreign Language Enjoyment (FLE) has been widely investigated in English learning, Chinese as a Foreign Language Enjoyment (CFLE) remains underexplored, particularly in AI-supported culturally immersive contexts. Guided by Self-Determination Theory (SDT), this study examines how an AI-supported immersive Chinese culture course contributes to learners’ psychological needs and the experience of CFLE among international students in China.

**Methods:**

A sequential explanatory mixed-methods design was used. Eighty-two international students joined the study completing a 40-item questionnaire assessing teacher-, learner-, and environment-related CFLE. Reliability and validity analyses, descriptive statistics, and Pearson correlation analyses were performed. Eight participants completed semi-structured interviews, and the interview data were analyzed thematically.

**Results:**

Participants reported moderately high levels of CFLE (*M* = 3.59–3.71, *SD* = 0.748–0.887). Reliability and validity analyses indicated satisfactory internal consistency, with Cronbach’s *α* = 0.951. Pearson correlations revealed strong positive relationships among teacher-, learner-, and environment-related enjoyment (*r* = 0.743–0.805, *p* < 0.01), suggesting that CFLE functions as an integrated affective system. Thematic analysis showed that enjoyment was exprienced through culturally responsive teaching, learner competence development, authentic cultural immersion, and AI-supported teacher-mediated learning, despite inadequately explained AI feedback occasionally leading to frustration or self-doubt.

**Discussion:**

Interpreted through SDT, CFLE appeared to surface in learner’s reported accounts when cultural immersion and AI-supported instruction was perceived as jointly satisfying learners’ needs for autonomy, competence, and relatedness. The findings advance CFLE research and suggest implications for the design of AI-supported immersive CFL learning environments.

## 1 Introduction

Emotions play a central role in second language acquisition (SLA), shaping learners’ engagement, persistence, and achievement (MacIntyre, 2016; Dewaele and Li, 2022). While some research has predominantly focused on negative emotions, such as foreign language anxiety (e.g., Ye et al., 2026; Aljohani and Mugaddam, 2025; Attia and Algazo, 2025; Chen et al., 2022) and demotivation (Peng and Lou, 2025), the emergence of Positive Psychology has redirected scholarly attention toward the facilitative role of positive emotions in language learning (MacIntyre et al., 2019). Among these, Foreign Language Enjoyment (FLE) has become one of the most extensively investigated constructs in contemporary SLA research (e.g., Dewaele and MacIntyre, 2014; Dewaele et al., 2023b; Dewaele et al., 2023a; Dewaele and Alfawzan, 2018; Kim, 2024). Defined as the positive emotional experience that arises when learners successfully engage with challenging language-learning tasks in supportive learning environments (Botes et al., 2022b; Dewaele and MacIntyre, 2014), FLE has consistently been associated with greater motivation, engagement, and language achievement (Botes et al., 2022a; Dewaele et al., 2023a; Moskowitz and Dewaele, 2020; Dewaele et al., 2023b). Rather than merely reflecting successful learning outcomes, enjoyment functions as an important motivational resource that sustains learners’ long-term involvement in language learning (Dewaele et al., 2023b; Saito et al., 2018; Dewaele and MacIntyre, 2014).

The study of FLE is particularly relevant in the context of Chinese as a Foreign Language (CFL) education. As China’s internationalisation of higher education continues to expand, increasing numbers of international students, particularly students from Belt and Road partner countries, are pursuing studies at universities in China. Unlike learners studying Chinese in their home countries, international students in China are immersed in authentic yet unfamiliar linguistic and cultural environments that provide continuous opportunities for real-world communication, intercultural interaction, and participation in local communities, as well as ongoing challenges. Such experiences extend language learning beyond the classroom and create distinctive conditions for emotional engagement. Emerging evidence suggests that authentic engagement with target-language communities can promote enjoyment by enabling learners to experience language as a living medium of communication and cultural participation rather than merely an academic subject (Duff, 2019; Hwang et al., 2024).

Meanwhile, advances in artificial intelligence (AI) are transforming language education. Recent studies have shown that AI-supported instruction with adaptive learning pathways, personalised feedback, multimodal learning resources, and real-time instructional support can foster positive affective experiences when meaningfully integrated into pedagogical practice (Huang et al., 2022; De la Vall and Araya, 2023; Wang F. et al., 2024). While culturally immersive programmes emphasize experiential learning and social participation, AI applications have predominantly been used to support discrete language skills such as vocabulary acquisition, reading skills, and grammar development (AlTwijri and Abdelhalim, 2026; Crompton et al., 2024). Consequently, little is known about how AI-supported culturally immersive experiences may work together to influence learners’ emotional experiences.

A similar limitation can be observed in existing FLE research, which has largely examined antecedents of enjoyment in isolation, focusing on factors such as teacher mediation, peer interaction, classroom atmosphere, or technology-supported learning environments separately (Botes et al., 2021; Dewaele and MacIntyre, 2014; Hosseini et al., 2022; Lin and Wang, 2025). Although these studies have identified important sources of positive emotion, they offer limited insight into how multiple learning affordances jointly contribute to the profile of enjoyment. Self-Determination Theory (SDT; Ryan and Deci, 2020, 2024) provides a useful lens for interpreting these processes. According to SDT, positive educational experiences are more likely to occur when learners perceive support for their needs for autonomy, competence, and relatedness (Noels et al., 2019; Ryan and Deci, 2020, 2024). From this perspective, AI-supported culturally immersive learning may foster learners’ autonomy, competence, and relatedness, thereby promoting CFLE in distinctive ways. Although the present study does not seek to test SDT directly, it adopts the theory as an interpretive lens for understanding how different instructional affordances may contribute to learners’ CFLE.

Against this backdrop, the present study explores international students’ experiences of CFLE in a Chinese culture course that integrates AI support with structured cultural activities from an emotion-motivation perspective.

## 2 Literature review

### 2.1 Foreign language enjoyment and its expansion into CFLE

As one of the most extensively investigated constructs in second language acquisition (SLA), the conceptualization of FLE has evolved from a predominantly individual-level perspective to a multidimensional understanding of enjoyment as a socially and contextually situated phenomenon. Early FLE research primarily examined the influence of teachers, peers, and classroom atmosphere on learners’ emotional responses (Dewaele and MacIntyre, 2014). To capture these experiences more systematically, researchers subsequently developed and validated a series of FLE measurement instruments. Dewaele and MacIntyre (2014) first introduced a 29-item foreign language enjoyment measurement. Subsequent principal component analysis led to a refined 14-item version (Dewaele and MacIntyre, 2016). Building on this work, Botes et al. (2021) further developed the Short Form of the Foreign Language Enjoyment Scale (S-FLES), a psychometrically robust 9-item instrument.

In the Chinese context, Li et al. (2018) validated a Chinese version of the FLE scale using a large sample of secondary-school English learners (*N* = 2,078) and identified three interrelated dimensions: FLE-Teacher, FLE-Private, and FLE-Atmosphere. This multidimensional framework has subsequently been widely adopted in EFLE research and has provided important evidence that enjoyment is co-constructed through interactions among learners, teachers, and learning environments.

In addition to the conceptualization of the FLE construct, recent reviews indicate that FLE has been examined across diverse educational settings and learner populations (Çobanoğulları et al., 2025; Dewaele and MacIntyre, 2014; Jiang and Dewaele, 2019; Staedtler et al., 2025; Tang et al., 2024; MacIntyre and Vincze, 2017; Nakanishi and Takeuchi, 2024). However, the majority of existing studies have focused on EFL contexts, while comparatively less attention has been devoted to CFL learners (Zhang Z. et al., 2024). As China continues to attract increasing numbers of CFL international students (Wu et al., 2021), understanding how positive emotions are experienced and sustained in CFL learning contexts has become an important area of inquiry.

CFLE shares core features with FLE as conceptualized by Dewaele and MacIntyre (2014), namely, positive emotional engagement in language learning, not limited to linguistic form-focused instruction but encompasses learners’ emotional experiences in foreign language contexts broadly construed, including culturally embedded immersive and communicative experiences. Due to the specific nature of CFL, CFLE also possesses distinctive characteristics, including (a) the logographic writing system which presents unique orthographic challenges and rewards; (b) greater cultural and linguistic distance for learners from Central Asian and African backgrounds compared with EFLE. Drawing on Li et al. (2018), rather than assuming generalizability from EFL-based FLE research, the present study therefore treats CFLE as a context-sensitive construct that warrants separate empirical investigation when enjoyment may emerge from the dynamic interplay among teacher mediation, learner agency, technological affordances, and culturally immersive environments.

### 2.2 Emotional–motivational impact of cultural immersion and AI-supported instruction

Positive emotions in language learning emerge from the broader sociocultural ecology of language use rather than solely from the classroom environment. Cultural immersion, defined as sustained participation in the communicative, social, and cultural practices of the target language community, has consistently been associated with enhanced motivation, intercultural competence, identity development, and emotional engagement (Spenader et al., 2018; Duff, 2019). Within CFL education, where language and culture are widely regarded as inseparable dimensions of learning (Jackson, 2023), immersion enables learners to participate in authentic cultural practices rather than merely acquire linguistic knowledge, thereby fostering enjoyment, a sense of belonging and integrative motivation (Dewaele et al., 2023b; Dewaele et al., 2023a; Gardner, 2010; Levy-Keren et al., 2024; Zhao, 2022). However, the emotional benefits of immersion are not uniform. The quantity and quality of intercultural contact, opportunities for meaningful social interaction, and learners’ individual adaptability all shape the extent to which immersion promotes positive emotions and sustained engagement (Duff, 2019; Jackson, 2023). Consequently, further research is needed to identify the pedagogical and contextual conditions under which cultural immersion may most effectively cultivate positive emotional experiences in CFL learning.

AI-supported instruction offers a complementary learning ecology. Intelligent tutoring systems, chatbots, and adaptive platforms provide personalised feedback, self-paced learning pathways, and multimodal resources (Deng et al., 2024; Huang et al., 2022; Lee and Lee, 2024; Wang D. et al., 2024; Zawacki-Richter et al., 2019). Affectively, AI can reduce learners’ fear of negative evaluation by providing a psychologically safer environment for linguistic experimentation, thereby promoting enjoyment (Dewaele and MacIntyre, 2014; Wu T. T. et al., 2025; Wu X. et al., 2025; Klimova and Pikhart, 2025). Its immediate, non-judgmental feedback also supports learners’ needs for competence and autonomy, consistent with Self-Determination Theory (Ryan and Deci, 2020; Wu T. T. et al., 2025; Wu X. et al., 2025). Recent research by Zhang et al. (2026) further corroborates this trend, identifying distinct motivation and enjoyment profiles among Chinese university students engaged in GenAI-assisted academic writing, thereby underscoring the salience of positive affective states in AI-supported language learning contexts. However, AI-supported interaction cannot fully replace emotional reciprocity, spontaneous negotiation of meaning, and authentic interpersonal relationships (Smith et al., 2025; Wang F. et al., 2024). Consequently, the affective value of AI might largely depend on how thoughtfully it is integrated into human-centred pedagogy rather than on AI support itself.

These two ecologies, immersion grounded in sociocultural participation and AI in technological mediation, potentially interact synergistically (Zhi and Wu, 2023). Yet research has examined them largely in isolation. What remains unexamined is how learners emotionally navigate environments where both coexist, and whether their combination produces benefits exceeding their separate effects. This gap directly motivates the present study’s first two research questions: identifying the overall levels of CFLE in an integrated setting (RQ1) and determining how AI-supported immersive activities contribute to CFLE (RQ2).

### 2.3 Self-determination theory and a framework of CFLE interpretation

To interpret the emotional-motivational experience of cultural immersion and AI-supported instruction, the study draws upon Self-Determination Theory (SDT), which posits that autonomy, competence, and relatedness are essential psychological needs whose satisfaction cultivates intrinsic motivation and well-being (Ryan and Deci, 2020). In language learning, need satisfaction predicts motivation, engagement, and enjoyment (Grenier et al., 2024; Noels et al., 2019).

Cultural immersion provides a rich context for learners’ psychological need satisfaction. Learners exercise autonomy through self-directed discovery, develop competence via authentic communicative success, and forge relatedness through genuine social bonds (Ryan and Deci, 2020; Yang, 2024; Wu and Dong, 2024). Yet immersion may also thwart needs: limited proficiency may constrain autonomy, communicative breakdowns may undermine competence, and social isolation may threaten relatedness (Noels et al., 2019). The emotional valence of immersion thus emerges from a dynamic balance between need satisfaction and thwarting.

AI-supported instruction presents a distinctive motivational profile (Wu X. et al., 2025; Zhang C. et al., 2024). Learners’ autonomy is enhanced through self-paced learning, personalised learning pathways, and opportunities for independent practice, while competence is fostered through immediate, adaptive, and non-judgmental feedback that supports incremental mastery (Huang et al., 2022; Li et al., 2025). However, despite these pedagogical affordances, AI-supported interaction remains qualitatively different from human interpersonal communication (Zhou and Li, 2026). The predominantly individualistic nature of AI engagement may therefore limit the satisfaction of learners’ need for relatedness, particularly when opportunities for genuine human interaction are reduced (Jeon et al., 2023; Li et al., 2025). Consequently, although AI effectively supports autonomy and competence, its affective benefits may depend on thoughtful integration with teacher mediation and collaborative learning experiences that sustain authentic social connection.

An integrated environment combining both modalities may thus enable synergistic need satisfaction. This informs RQ3, which asks how CFLE experience in this environment can be interpreted through SDT, specifically, how patterns of autonomy, competence, and relatedness satisfaction explain why particular configurations of cultural immersion and AI support result in particular emotional-motivational outcomes among CFL learners.

## 3 Methodology

### 3.1 Research questions and research design

Building on the literature reviewed above, this study investigates CFLE among international students in an AI-supported culturally immersive learning environment and is guided by the following research questions:

RQ1: What are the levels of the teacher-, learner-, and environment-related CFLE among international students enrolled in an AI-supported immersive Chinese culture course?

RQ2: How do AI-supported culturally immersive activities and learning experiences contribute to the teacher-, learner-, and environment-related CFLE?

RQ3: How can the experience of CFLE in this integrated learning environment be interpreted through the lens of Self-Determination Theory (SDT), particularly in relation to learners’ needs for autonomy, competence, and relatedness?

To address these research questions, this study employed a sequential explanatory mixed-methods design (Creswell and Plano Clark, 2017; Fetters and Tajima, 2022; Guo, 2021). Quantitative data were collected and analyzed first to examine the levels of CFLE, followed by qualitative interviews designed to explain and elaborate the survey findings. Integration occurred both during the development of the interview and in the Results and Discussion sections, whereby the quantitative results informed the interview focus, and the qualitative findings were subsequently used to explain, contextualize, and enrich the statistical patterns.

Specifically, the questionnaire data addressed RQ1 by examining CFLE levels. The interview data addressed RQ2 by providing in-depth accounts of learners’ lived experiences. Finally, the integration of both datasets, interpreted through the SDT framework, addressed RQ3 by explaining the psychological mechanisms underlying CFLE formation. Given the absence of statistically meaningful between-group variation and the explanatory nature of the data, RQ3 focuses on SDT-informed interpretation of CFLE experience rather than nationality-based comparison.

### 3.2 Research context

The study was conducted in an AI-supported immersive Chinese culture course designed for second-year international undergraduate students at a university in a major city of eastern China.

For this study, AI- supported instruction is defined as algorithm- driven pedagogical practice featuring dynamic generation, intelligent feedback and adaptive responses, which is conceptually separated from non- intelligent digital technologies including VR, digital libraries, static visualisation, and conventional gamified platforms. These general digital tools only deliver pre- built content, resources or fixed rule- based activities without interpreting learner input or tailoring outputs.

This course unfolded over 16 weeks and was taught sequentially by three different teachers. Unlike the previous lecture-based culture course delivered by teachers with chalk and PowerPoint (Tsou, 2005; Spenader et al., 2018), this course systematically embedded AI technologies into the instructional design to support language and culture learning. Weeks 1–4 with Ms. A employed AI animation (not pre- rendered clips), where learners requested visual adjustments to unpack core cultural concepts. Weeks 5–10 under Ms. B used static visualisation and traditional Chinese medicine (TCM) simulations as non- AI background resources; embedded AI functions offered real- time argument prompts and personalised feedback during gamified historical debates. Weeks 11–16, Ms. C had students utilise AI analytical functions for digital metrology and data- driven cultural analysis. Rather than passively consuming digital materials, learners actively prompted, revised and interpreted outputs produced by AI components throughout each phase. Crucially, these technologies were not deployed in isolation but were instead integrated into pedagogically structured learning tasks, the preponderance of which were classroom-based. Although embedded within the instructional setting, they did not constitute the primary focus of the present investigation.

Meanwhile, the cultural content (e.g., Hanfu culture, TCM, tea trade) is used as an authentic and immersive pedagogical medium to facilitate language-related engagement and interaction. Complementing the AI-supported immersive instruction delivered in Rooms 302 and 401 equipped with high-performance computing facilities and virtual reality devices (e.g., RTX 4080 GPUs and VR headsets), the course integrated Chinese learning with a range of structured culturally immersive activities, including community-based experiences (e.g., Bainaohui PC Mall, Dream Creative Town, and local markets), visits to heritage sites (e.g., Hefangjie Pedestrian Street). These activities were designed to foster authentic engagement with Chinese culture.

As a result, students’ learning experiences extended across physical-digital-social-cultural domains over the 16-week period, forming a hybrid learning ecology that combined cultural immersion with AI-supported instruction, which provided an empirical setting for this inquiry.

### 3.3 Participants

A total of 82 international students enrolled in the course participated in this study (Table 1), with data collected via a convenience sampling method (Peterson and Merunka, 2014). The participants included 38 female and 44 male students aged 19 to 22 from Morocco, South Africa, Kazakhstan, Kyrgyzstan, Tajikistan, Turkmenistan, and Uzbekistan, etc., demonstrating somewhat diverse linguistic, educational, and cultural backgrounds. They had attended a one-year Chinese language programme and obtained considerable Chinese proficiency in speaking, listening, writing, and reading at HSK Level 4.

**Table 1:** Information of the participants (*N* = 82).

Chinese language level	Intermediate		Total	
	*n*	%	*n*	%
Female	38	46	38	46
Male	44	54	44	54

### 3.4 Instruments

#### 3.4.1 Questionnaire development

The questionnaire was adapted from the validated Chinese version of the Foreign Language Enjoyment (FLE) scale (Li et al., 2018). While the original instrument has demonstrated strong reliability and validity in prior research, it was originally designed for secondary-level English learners and does not fully capture the complexity of CFLE involving AI-supported culturally immersive learning in higher education contexts.

To address this limitation, additional items were designed to capture learners’ experiences across three interrelated domains: (1) culturally immersive activities, (2) AI-mediated learning support, and (3) study-abroad contextual affordances. These items were intended to capture the contextual conditions conducive to CFLE, rather than affective responses alone. Item development was informed by prior FLE research (Çobanoğulları et al., 2025; Dewaele and MacIntyre, 2014; Li et al., 2018), CFL pedagogical literature, and systematic observation of the focal course.

All newly developed items underwent expert review by experienced CFL instructors and the research team to establish content validity, clarity, and theoretical alignment with the CFLE framework.

The final CFLE questionnaire comprised 40 items which were organized into three explanatory theoretical categories, teacher-, learner-, and environment-related CFLE, derived from the theoretical literature (Li et al., 2018). The categories provided a structured framework to guide item construction and descriptive reporting, yet they were not intended to represent empirically validated latent dimensions or subscales.

All items were measured on a five-point Likert scale ranging from 1 (strongly disagree) to 5 (strongly agree), with higher scores indicating higher levels of CFLE. Prior to the main data collection, a pilot study was undertaken to assess the clarity and logical flow of the questionnaire items (Malmqvist et al., 2019). Reliability analyses subsequently indicated satisfactory internal consistency for the overall scale and its constituents.

#### 3.4.2 Interview protocol

A semi-structured interview protocol was developed based on Li et al.’s (2018) three-category framework of FLE. The protocol was linguistically reviewed and refined to ensure clarity, cultural appropriateness, and interpretive consistency for participants with diverse linguistic backgrounds.

### 3.5 Interview procedures

Following the questionnaire survey, a subsample of 8 participants with an intermediate level of Chinese (HSK Level 4) was selected from volunteers via maximum variation sampling to capture heterogeneity across gender composition (3 males and 5 females) and academic discipline (balanced at 4 Business Chinese and 4 Computer Science). This sampling approach enabled systematic cross-case comparison while ensuring diversity relative to the full participant pool (*N* = 82). The profiles of 8 participants are presented, with their pseudonyms adopted to ensure confidentiality (Table 2).

**Table 2:** Profile of interview participants (*N* = 8).

Participant	Pseudonym	Gender	Nationality	HSK	Major
P1	Aida	Female	Kazakhstan	Level 4	BC
P2	Jamshed	Male	Uzbekistan	Level 4	CS
P3	Nadir	Male	Tajikistan	Level 4	CS
P4	Aisha	Female	Kyrgyzstan	Level 4	BC
P5	Berdi	Male	Turkmenistan	Level 4	CS
P6	Zulhumar	Female	Uzbekistan	Level 4	BC
P7	Aliya	Female	Kazakhstan	Level 4	CS
P8	Fatima	Female	Morocco	Level 4	BC

BC, Business Chinese; CS, Computer Science.

To triangulate survey findings, semi-structured interviews were conducted (Dewaele and MacIntyre, 2014; Guo, 2021; Wang Z. et al., 2024). Each 30-min interview followed a protocol systematically addressing three enjoyment components: teacher-, learner-, and environment-related CFLE. Particular emphasis was placed on understanding how cultural immersion and AI-supported instruction jointly shaped learners’ emotional engagement with CFL learning. Interviews were audio-recorded, transcribed verbatim, and iteratively refined through member checking to ensure accuracy.

### 3.6 Measurement validation

Prior to addressing the research questions, the psychometric properties of the data from the CFLE survey were examined.

Firstly, the data were analyzed to show the profile of the 40 questionnaire items (Table 3). Both the skewness (−1.152 to + 0.241) and kurtosis (−1.366 to + 0.442) values fell within the acceptable range of −2 to +2 (George and Mallery, 2010), indicating that the data approximate a normal distribution and are suitable for further analysis. Corrected item-total correlations (CITCs) ranged from 0.259 (Q9) to 0.762 (Q15). Although four items slightly fell below the 0.30 threshold, the deletion would not substantially improve reliability (*Δα* ≤ 0.001), confirming that the instrument is reliable and valid for further examining CFLE in this sample.

**Table 3:** Descriptive statistics for participants’ questionnaire responses (*N* = 82).

Item	Min	Max	Mean	SD	Skew ness	Kurtosis	CITC
Q1	1	5	3.35	1.261	−0.286	−0.856	0.36
Q2	1	5	3.62	0.982	−0.215	−0.578	0.439
Q3	1	5	3.41	1.247	−0.447	−0.598	0.265
Q4	1	5	3.37	1.257	−0.278	−0.899	0.513
Q5	1	5	3.73	1.248	−0.644	−0.509	0.597
Q6	1	5	4.05	1.142	−1.054	0.235	0.623
Q7	1	5	3.49	1.389	−0.461	−1.022	0.702
Q8	1	5	3.59	1.263	−0.405	−1.059	0.54
Q9	1	5	3.3	1.277	−0.063	−1.114	0.259
Q10	1	5	3.8	1.213	−0.616	−0.6	0.37
Q11	1	5	3.83	1.202	−0.676	−0.651	0.522
Q12	1	5	4.04	1.152	−1.093	0.442	0.638
Q13	1	5	3.95	1.254	−0.997	0.011	0.737
Q14	1	5	3.7	1.316	−0.483	−1.132	0.719
Q15	1	5	3.6	1.18	−0.258	−1.102	0.762
Q16	1	5	3.74	1.233	−0.637	−0.565	0.705
Q17	1	5	3.56	1.151	−0.316	−0.825	0.485
Q18	1	5	3.79	1.293	−0.725	−0.6	0.641
Q19	1	5	3.65	1.257	−0.553	−0.663	0.631
Q20	1	5	3.36	1.07	0.241	−0.669	0.46
Q21	1	5	3.88	1.241	−0.835	−0.463	0.618
Q22	1	5	3.84	1.202	−0.65	−0.838	0.749
Q23	1	5	3.71	1.252	−0.738	−0.354	0.712
Q24	1	5	3.76	1.161	−0.515	−0.834	0.711
Q25	1	5	3.38	1.241	−0.449	−0.616	0.547
Q26	1	5	3.62	1.146	−0.423	−0.723	0.643
Q27	1	5	3.51	1.259	−0.332	−1.014	0.637
Q28	1	5	3.68	1.273	−0.599	−0.689	0.567
Q29	1	5	3.78	1.14	−0.534	−0.548	0.649
Q30	1	5	3.63	1.16	−0.359	−0.633	0.599
Q31	1	5	3.49	1.333	−0.457	−0.883	0.276
Q32	1	5	2.89	1.467	0.058	−1.366	0.295
Q33	1	5	3.66	1.362	−0.652	−0.786	0.668
Q34	1	5	4.04	1.251	−1.152	0.229	0.503
Q35	1	5	3.47	1.107	−0.185	−0.67	0.604
Q36	1	5	3.41	1.313	−0.423	−0.834	0.509
Q37	1	5	4.09	1.145	−1.115	0.362	0.679
Q38	1	5	3.19	1.469	−0.168	−1.276	0.452
Q39	1	5	3.82	1.295	−0.809	−0.58	0.529
Q40	1	5	3.25	1.102	0.096	−0.52	0.498

The reliability of the 40-item questionnaire data was also examined through Cronbach’s *α* (Table 4). It is found that the scale yielded an overall Cronbach’s *α* coefficient of 0.951 (higher than 0.826, Li et al., 2018), with respective *α* coefficients of 0.825, 0.888, and 0.912 for the three categories, all of which exceeded the threshold for good reliability, indicating satisfactory internal consistency of the scale.

**Table 4:** Reliability tests for the CFLE questionnaire.

Test	Value	Interpretation
Cronbach’s *α* (Overall)	0.951	>0.7
Cronbach’s *α* (Environment-related items)	0.825	>0.7
Cronbach’s *α* (Teacher-related items)	0.912	>0.7
Cronbach’s *α* (Learner-related items)	0.888	>0.7

Cronbach’s α and CITC values indicate internal consistency within each category; they do not seem to establish factorial distinctiveness. Category-level statistics are reported here for descriptive transparency only.

### 3.7 Data analysis

The mixed-methods study integrated quantitative data with qualitative data.

The quantitative questionnaire data were analyzed using SPSS 26.0. Descriptive statistics (means, standard deviations, skewness, and kurtosis), CITC, and Cronbach’s *α* were computed to assess item properties and reliability. To address RQ1, mean and SD scores were calculated for the three categories of CFLE. Pearson correlation analyses were then performed to explore relationships among the three categories.

For the qualitative strand, semi-structured interviews informed by the priori framework of teacher-, learner-, and environment-related CFLE were transcribed verbatim and analyzed using inductive thematic analysis (Braun and Clarke, 2021). The analysis followed the six-phase procedure: familiarization with the data, systematic coding, theme generation, theme review, refinement and definition, and report production. Two researchers independently coded 20% of the transcripts using a deductive-inductive thematic analysis framework (Braun and Clarke, 2021). Inter-coder agreement was assessed using Cohen’s kappa, which yielded *κ* = 0.84, indicating ‘almost perfect’ agreement (Landis and Koch, 1977). Disagreements were resolved through discussion until consensus was reached; where consensus could not be reached, a third researcher adjudicated. The remaining 80% of transcripts were coded by one researcher, with periodic checks against the established coding frame to ensure consistency. The deviant or contradictory cases during coding and theme development were also identified to verify interpretation accuracy. For RQ2, the resulting themes and codes supported by participant excerpts, is presented in the Results section, while theoretical interpretation using SDT is provided in the Discussion.

To further integrate the two strands, the research team convened to construct the joint display, using the quantitative profiles to guide the selection of qualitative themes and excerpts to interpret quantitative anomalies. This process was iterative: initial displays were refined as new integrative insights emerged, and some displays were discarded when the integration added no interpretive value beyond what either source provided alone.

To address RQ3, the quantitative and qualitative data were integrated through SDT (Ryan and Deci, 2020), focusing on learners’ experiences of autonomy, competence, and relatedness in relation to CFLE. Qualitative themes were subsequently used to explain and enrich the quantitative results, interpreting from SDT to demonstrate how CFLE was constructed by explaining the motivational processes underlying learners’ experiences.

## 4 Results

### 4.1 Quantitative results: levels of CFLE

Descriptive statistics were computed, and Pearson correlation analyses were conducted to uncover the levels of CFLE (Table 5).

**Table 5:** Descriptive statistics and Pearson correlations for CFLE (*N* = 82).

Variable	M	SD	1	2	3
1. Environment	3.59	0.748	1		
2. Teacher	3.71	0.887	0.802^**^	1	
3. Learner	3.60	0.772	0.743^**^	0.805^**^	1

*p < 0.05, **p < 0.01.

The overall CFLE scale yielded a mean of 3.63 (average of 3.59, 3.71, 3.60) on the 5-point Likert scale, falling in the moderately high range. This indicates that participants, on average, reported a positive level of enjoyment in their CFL learning experience, reflecting a generally favorable attitude toward CFL among the sample.

Across the three categories, mean scores ranged from 3.59 to 3.71, all above the scale midpoint of 3.0, further confirming the positive learning enjoyment of the sample. Teacher-related category received the highest mean score (*M* = 3.71, *SD* = 0.887), indicating that teaching style, teacher-student interaction, and teacher mediation were perceived as the most positive aspects of the learning experience. It also showed the relatively high variability, suggesting more diverse learner perceptions. Learner-related category received the second mean score (*M* = 3.60, *SD* = 0.772), indicating that learners viewed their own engagement, interest, and cultural integration positively. Environment-related category ranked the lowest (*M* = 3.59, *SD* = 0.748), though still moderately high, reflecting generally positive evaluations of the environment, learning resources, and instructional materials with relatively consistent responses, albeit less favorably than teacher- and learner-related category.

Standard deviations (SD) across all categories ranged from 0.748 to 0.887, all within the moderate range (0.7–1.0). This indicates that there was sufficient variability in participants’ responses to capture meaningful individual differences in CFLE levels, without extreme homogeneity that would limit the interpretability of subsequent statistical analyses.

Pearson correlation coefficients were calculated to examine the bivariate relationships between the three CFLE categories, with the results providing critical evidence for the scale’s construct consistency and the interrelatedness of the CFLE categories.

All three CFLE categories exhibited strong, positive, and statistically highly significant correlations with each other (*r* = 0.743–0.805, *p* < 0.01). The strongest correlation was observed between learner-related and teacher-related category(*r* = 0.805, *p* < 0.01), indicating that participants who perceived a more positive learning enjoyment also reported significantly higher levels of enjoyment related to their teachers. The correlation between environmental-related and teacher-related category was *r* = 0.802 (*p* < 0.01), suggesting that a positive learning environment was strongly associated with greater enjoyment related to their teachers. Environment-related and learner-related category were correlated (*r* = 0.743, *p* < 0.01), the weakest among the inter-category correlations, but still in the strong positive range, reflecting that positive environment experiences were closely linked to participants’ intrinsic motivation and engagement in Chinese learning.

These strong inter-category correlations confirmed the construct coherence of the CFLE scale: all three categories converged on the overarching construct of CFLE. These results provided further evidence of the scale’s high internal consistency: each category was strongly and reliably associated with the overall CFLE construct.

In summary, the descriptive statistics and correlation analyses yielded two core conclusions for this study: (1) Participants in this sample reported moderately high levels of CFLE, with teacher-related CFLE rated as the most positive aspect of their learning experience, followed by the learner category and the environmental category. (2) The CFLE scale demonstrated satisfactory internal consistency and convergence: the three categories were strongly interrelated and were adequately representative of overall CFLE.

Although the three categories were derived from the FLE literature (Li et al., 2018) as an organizational framework for item development, their high intercorrelations (*r* = 0.743–0.805) indicated substantial overlap, suggesting that learners in this sample may have experienced CFLE as a unitary rather than differentiated affective state. We therefore treated them as explanatory groupings, not empirically distinct constructs. Whether these categories can be discriminated in other contexts or with refined items awaits future research.

### 4.2 Qualitative results: source of CFLE

A total of 18 codes are identified and organized into three themes (Table 6). To complement the quantitative findings, themes are illustrated with representative excerpts as follows.

**Table 6:** Themes and codes.

Themes	Codes
Teacher	Teacher mediating AI-supported cultural immersion
	Teacher’s explanations connecting AI operations with cultural understanding
	Teacher validating AI-supported cultural competence
	Teacher continuity in sustaining AI-supported learning
Learner	Emotional engagement through AI-supported cultural agency
	Experiences resembling flow in AI-culture synchronization
	Identity transformation: from language learner to cultural practitioner
	Challenges associated with AI-supported learning
Environment	Classroom atmosphere *Classroom atmosphere as collective AI-cultural immersion* *Group work as joint AI-cultural creation* *A supportive peer climate of AI-cultural assistance*
	Physical and institutional context *Infrastructure and technology sustaining AI-Cultural immersion* *Library and urban resources extending AI-Cultural practice* *Local communities as authentic AI-cultural transfer sites*
	AI affordances as culturally situated learning tools *AI visualization and immersion: dissolving historical distance* *AI gamification: sustained cultural role-play* *AI immediate feedback: culturally embedded accuracy*
	Positive perceptions of assessment

#### 4.2.1 One: teacher

In terms of “teacher mediating AI-supported cultural immersion,” the AI–cultural hybrid experiences was expressed. For example, we heard “*Instead of first explaining the text in the textbook, Ms. B asked us to open the ‘3D Hanfu Digital Archive’ that she had prepared in advance*” (Aida), and “*The classroom was arranged as a ‘Tang Dynasty marketplace in H city,’ while the projection screen displayed an AI-generated scene of a tea market by the Q River where each of us held a ‘Tang merchant identity card’*.” (Aisha).

Similarly, participants described teacher’s explanations connecting AI operations with cultural understanding. For example, participants noted: “*Ms. B said, ‘*xuéwèi, *see this point? Pressing it can help relieve a headache—that’s what an acupuncture point is*.’” (Aida), “shìbósī, *You can think of the Tang Dynasty Maritime Trade Office as the equivalent of today’s Customs Office in H city*.” (Aisha), “*You can think of bù (‘step’) as a basic unit in computer science*.” (Jamshed), and “píwèi, *the spleen and stomach are like the kitchen in a home: raw food is transformed into cooked nourishment, which in turn supplies the energy the body needs*.” (Zulhumar).

“The teacher validating AI-supported cultural competence” was also expressed. For example, we heard: “*Ms. B praised my outfit design as ‘being consistent with Tang Dynasty aesthetics’*” (Aida), “*Ms. B praised me for successfully integrating my academic major with Chinese culture*.” (Jamshed), and “*Ms. B said, ‘Aisha, your ability to capture the logic and elegance of traditional Chinese medicine in English is remarkable: you are more than ready to work as a translator in this field.’*” (Aisha).

Finally, teacher continuity for the three-sectioned course in sustaining AI-supported cultural learning experiences was highlighted. Participants consistently reported that changes in instructors did not diminish their enjoyment. For example, “*Rather than disrupting my learning, changing teachers enriched the course by exposing me to different AI-supported teaching approaches.*” (Aida), and “*Changing teachers did not diminish my enjoyment; instead, it helped build a more complete learning experience*.” (Aliya).

Taken together, participants consistently portrayed teachers as the central mediators connecting AI tools, cultural content, and meaningful learning experiences. This teacher-related experiences aligns with the quantitative finding that teacher-related CFLE received the highest mean score among the three categories (*M* = 3.71, *SD* = 0.887).

#### 4.2.2 Two: learner

In terms of “emotional engagement through AI-supported cultural agency,” the experience of performing culturally meaningful roles through AI was expressed. For example, we heard: “*Personally, operating it felt like stepping into a clothing shop in ancient China, surrounded by historical costumes and cultural atmosphere,*” (Aida), and “*I was completely immersed in the role of a Tang Dynasty merchant. Chinese was no longer text in a textbook, but a tool with which I could ‘make money’*” (Aisha).

Experiences resembling flow (Csikszentmihalyi, 1990) in AI-culture synchronization were expressed. For example, one participant described: “*My fingers precisely adjusted the parameters on the keyboard while my eyes were fixed on the screen, watching how the patterns matched while reading the Chinese prompts*.” (Aliya).

Identity transformation from language learner to cultural practitioner was expressed. For example, participants reported: “*Chinese is difficult, but AI makes it fun and useful*” (Aida), and “*Chinese is difficult, but combining it with computer science makes it meaningful and enjoyable*” (Jamshed). Jamshed, Nadir, Berdi, Aliya, and Fatima aimed to transform classroom wonder into commercial ventures inspired by entrepreneurial vision. “With *fluent Chinese, I plan to import Chinese tea and silk to Casablanca while returning artisanal Moroccan crafts to boutiques in H city after graduation*” (Fatima).

At the same time, challenges associated with AI-supported learning were expressed. Several learners indicated that AI systems sometimes identified errors without providing sufficient explanations, while others reported uncertainty regarding how to improve after receiving automated feedback. These experiences suggest that AI-supported learning was not uniformly positive and occasionally generated frustration or self-doubt.

Overall, learners described AI as enhancing agency, immersion, and cultural participation, despite presenting challenges related to feedback interpretation and self-regulated learning. These mixed experiences may help explain why learner-related CFLE received the second-highest mean score among the three (*M* = 3.60, *SD* = 0.772).

#### 4.2.3 Three: environment

For “classroom atmosphere as collective AI-cultural immersion,” shared participation in AI-supported cultural worlds was expressed. For example, we heard: “*Thanks to AI-cultural immersion, I successfully navigated a business negotiation entirely in Chinese*.” (Aida).

Group work as joint AI-cultural creation was expressed. Participants frequently emphasized collaborative engagement. For example, “*Throughout the whole process, we discussed everything in Chinese. Nobody felt tired or bored; instead, everyone found it interesting*.” (Aida).

A supportive peer climate of AI-cultural assistance was expressed. For example, Aisha explained: “*I did not need to worry about being laughed at for speaking poor Chinese; instead, I became more willing to speak voluntarily, especially during AI-supported simulations*.”

The role of infrastructure, technological resources and local communities in sustaining AI-cultural immersion was expressed. For example, participants noted: “*Room 302 had two large tables, the projection screen was very clear, and the air-conditioning temperature was just right*” (Aida), “*The computer lab environment in Room 401 had high-performance computers that ran very smoothly*” (Jamshed), and “*An aunt, vendor in Hefangjie Pedestrian Street, said that I spoke Chinese very well*” (Aisha).

AI affordances as culturally situated learning tools were expressed. For example, “*In the digital ‘weights-and-measures’ AI simulation system, when I entered ‘one dǒu of rice’, the AI generated a virtual ‘dǒu’ container*” (Jamshed), and “*In the AI-supported Chinese medicine consultation simulation, I assumed the role of a patient, describing symptoms such as headache and dry throat*.” (Fatima).

Positive perceptions of assessment were expressed. Participants generally believed that assessment tasks reflected authentic application of Chinese rather than rote memorization. For example, “*My grade reflected my real level. The examination process was not about ‘memorizing answers’, but about ‘using Chinese to do things’*” (Aida), while Berdi reported that “*AI-supported review activities helped maintain learning motivation after encountering difficulties.”*

Overall, participants perceived the learning environment as supportive of enjoyment through collaborative participation, immersive cultural activities, and AI-supported learning opportunities. However, environment-related CFLE received the lowest mean score (*M* = 3.59, *SD* = 0.748), indicating a numerically lower mean than that of teacher mediation and learner experiences.

### 4.3 Integration of quantitative-qualitative results

Integration in mixed-methods research requires more than confirming that two data sources yield similar conclusions (corroboration). It demands structured comparison that reveals how each source contributes distinct, non-redundant insights to the phenomenon under study (Fetters et al., 2013). Table 7 features a joint display synthesizing quantitative and qualitative findings to yield a holistic account of CFLE experience within the AI-supported cultural immersion curriculum. Teacher-related enjoyment received the highest mean score (*M* = 3.71), and the qualitative findings converged by explaining that teachers generated enjoyment through pedagogical mediation of cultural scaffolding and continuous guidance rather than through AI alone. Similarly, quantitative data concerning learner- and environment-related CFLE were elaborated through interview data, which revealed underlying mechanisms including AI-supported learner agency, flow-like engagement, identity development, collaborative classroom interaction, and authentic cultural participation. The integration also expanded the quantitative findings by identifying aspects that were not directly captured by the questionnaire, such as entrepreneurial aspirations and the role of AI in reducing psychological distance from Chinese culture. Moreover, qualitative evidence explained minor inconsistencies by showing that technological limitations occasionally constrained learner enjoyment. Overall, the joint display demonstrates complementarity between the two datasets, indicating that CFLE emerged from the dynamic interaction of teacher mediation, learner agency, AI affordances, and authentic cultural environments rather than from any single instructional component. These integrative moves transform the mixed-methods design from a sequential explanatory structure into a genuinely dialogic analysis, where quantitative and qualitative data interrogate and refine one another.

**Table 7:** Joint display of quantitative and qualitative findings on CFLE.

CFLE	Quantitative findings	Qualitative category	Integration interpretation
Teacher-related	*M* = 3.71, *SD* = 0.887 (highest); (*r* = 0.802–0.805 ** *p* < 0.01)	Teacher as mediator of AI-cultural immersion	Convergence and elaboration: high teacher scores are explained by teachers’ mediation of AI into meaningful cultural learning.
		Cultural scaffolding and feedback	Complementarity: teacher affirmation strengthened competence beyond AI-generated feedback.
Learner-related	*M* = 3.60, *SD* = 0.772	AI-supported agency and flow	Complementarity: enjoyment stemmed from learner agency and deep engagement that the survey measured only indirectly.
		Identity development and AI challenges	Expansion and discordance: CFLE included identity growth, while occasional AI errors explain slightly lower learner scores.
Environment-related	*M* = 3.59, *SD* = 0.748	Collaborative classroom and authentic contexts	Convergence: positive environmental ratings reflected collaborative participation and authentic cultural experiences.
		AI-supported immersion	Expansion: AI visualization and feedback increased immersion beyond what environmental survey items captured.
Overall	Teacher-, Learner-, and Environment-strongly correlated (*r* = 0.743–0.805 ** *p* < 0.01)	Cross-category synthesis	Meta-integration: CFLE was experienced through the interaction of teacher mediation, learner agency, AI affordances, and authentic cultural participation.

Quantitative findings are based on the CFLE questionnaire (*N* = 82), with Teacher- (M = 3.71, SD = 0.887), Learner- (M = 3.60, SD = 0.772), and Environment- (M = 3.59, SD = 0.748).

Qualitative findings derive from semi-structured interviews with 8 participants. Following Fetters et al. (2013), the joint display integrates the datasets through convergence, complementarity, expansion, discordance with explanation, and contextualization, demonstrating not only whether the quantitative and qualitative findings align but also how each dataset contributes unique insights into the experience of CFLE in an AI-supported immersive Chinese culture course.

## 5 Discussion

### 5.1 Levels of CFLE in culturally immersive and AI-supported learning

The findings indicated a moderately high level of CFLE (*M* = 3.59–3.71) and are broadly consistent with previous studies reporting moderate-to-high levels of foreign language enjoyment among learners of Chinese and English in tertiary educational settings (Dewaele and MacIntyre, 2014; Jiang and Dewaele, 2019; Li et al., 2018). However, the present findings complement and extend this literature by suggesting that enjoyment can be experienced in AI-supported immersive CFL settings despite the considerable linguistic and cultural demands associated with learning Chinese culture.

This finding is particularly noteworthy within the CFL context. Unlike learners of English, who are often exposed to the target language and culture through global media and international communication, CFL learners frequently encounter a linguistic and cultural system that is comparatively unfamiliar. Beyond linguistic cognitive demands, culturally specific communicative norms add significant cultural learning burdens for Chinese learners (Yang et al., 2025; Wang et al., 2023). Nevertheless, participants largely reported relatively high levels of enjoyment, suggesting that challenge and enjoyment are not mutually exclusive. Rather, positive emotions appeared capable of coexisting with learning difficulties, broadly supporting previous research that tends to identify enjoyment as an important psychological resource for engagement, persistence, and resilience in language learning (Dewaele and MacIntyre, 2014; Li et al., 2018).

Another important finding concerns the role of AI in shaping learners’ enjoyment. Research suggests that AI-supported learning may diminish emotional engagement due to challenges (Klimova and Pikhart, 2025). However, the present findings do not provide conclusive evidence for this assumption. Participants tended to report high levels of enjoyment despite extensive engagement with AI-supported immersive learning activities. This observation aligns with recent studies suggesting that AI can enhance learner engagement when integrated into meaningful pedagogical contexts (Chang and Sun, 2024; Wang F. et al., 2024). Thus, AI appeared to function less as a technological innovation and more as a supportive tool facilitating cultural participation. This finding suggested that AI was perceived as contributing to enjoyment indirectly through cultural engagement rather than through technological novelty alone.

A further important finding concerns the rating of CFLE. Teacher-related enjoyment received the highest rating, followed by learner-related enjoyment and environment-related enjoyment. This observed pattern aligns broadly with findings from prior research identifying teachers as major contributors to foreign language enjoyment (Dewaele et al., 2019; Dewaele et al., 2023a; Jiang and Dewaele, 2019). The teacher-related enjoyment may be particularly significant in CFL settings, where learners often encounter unfamiliar linguistic and cultural systems. Under such circumstances, teachers appeared to serve not only as instructors but also as cultural mediators who helped learners interpret cultural practices, understand social norms, and connected classroom learning with authentic aspects of Chinese society (Dewaele et al., 2023b; Feldman-Maggor et al., 2025). The findings therefore tentatively suggested that the emotional significance of teachers may extend beyond instructional support to cultural and intercultural mediation.

In contrast, learner-related enjoyment ranked the second after teacher-related CFLE in terms of mean score. One possible explanation might be that deriving enjoyment from one’s own performance may be perceived more challenging during the early stages of CFL learning, when learners have not yet accumulated sufficient linguistic competence or mastery experiences to support positive self-evaluations (Botes et al., 2022b; Jin and Zhang, 2021). Consequently, positive emotions may be perceived to initially depend more heavily on external sources of support, such as teachers and learning environments, than on internally generated feelings of competence (Feldman-Maggor et al., 2025). This observed pattern pointed to the possibility that enjoyment in CFL contexts may be mediated by social scaffolding before becoming increasingly internalized through the development of learner agency and self-confidence.

Participants generally associated CFLE with future entrepreneurial aspirations, expressing intentions to apply their Chinese language proficiency and cultural knowledge in ventures connecting China with their home countries. This thematic pattern tentatively suggested that CFLE may be perceived to have extended beyond classroom enjoyment, signifying meaningful cultural engagement and future-oriented motivation.

In summary, the findings suggested that CFLE in AI-supported CFL learning was perceived to emerge largely through the interaction of teacher mediation, culturally immersive activities, and learner participation. More importantly, they indicated that CFLE was perceived to be shaped not primarily by AI innovation itself but by the extent to which AI support facilitates meaningful engagement with the culture of the target language.

### 5.2 Learners’ perceived sources of CFLE in culturally immersive and AI-supported learning

The qualitative findings suggested that CFLE seemed to be strongly shaped by AI-supported, socially situated cultural engagement rather than AI exposure alone.

Teacher-related enjoyment appeared as the most salient. Consistent with established FLE scholarship (Dewaele and MacIntyre, 2014; Jiang and Dewaele, 2019; Li et al., 2021), participants attributed positive learning emotions mostly to supportive, encouraging teacher mediation. Beyond conventional instructional roles, teachers appeared to serve as essential cultural mediators. Navigating unfamiliar Chinese cultural concepts, including *xuéwèi* (acupoints), *píwèi* (spleen-stomach theory), and *shìbósī* (ancient dynastic customs offices), students tended to rely on teachers who generally mediated by contextualizing AI-generated content, clarifying cultural connotations, and bridging new knowledge with prior experience (Feldman-Maggor et al., 2025). It is largely through teacher mediation that AI support may transform into meaningful cultural learning. Extending prior literature, these results generally confirm that AI support is perceived as elevating, rather than diminishing, teachers’ irreplaceable cultural and pedagogical mediational functions.

Learner-related enjoyment also appeared to be salient. In AI simulations, learners tended to adopt culturally grounded roles as merchants, translators, and intercultural negotiators, entrepreneurs, shifting from passive knowledge recipients to active cultural participants. Such agentic participation was perceived as associated with a heightened sense of cultural connection and intercultural communication confidence. This aligns with Language Investment Theory (Darvin and Norton, 2015; Norton, 2013), supporting the notion that identity construction may underpin affective and behavioural engagement in culturally immersive CFL learning.

Despite its lowest quantitative rating, environment-related enjoyment seemed to reflect profound, identity-driven engagement. Students largely engaged in AI-supported immersive activities, such as virtual historical scenarios, digital cultural exhibitions, and culturally embedded role-plays, which appeared to foster intimate, embodied connections with the target culture (Han et al., 2025; Klimova et al., 2024). Many participants reported experiencing “*stepping into ancient Chinese life*” through virtual environments, which seemed to have narrowed learner-culture distance and converted abstract cultural knowledge into tangible immersive experiences. Unlike existing studies that largely link environmental enjoyment to classroom atmosphere and peer dynamics (Dewaele and MacIntyre, 2014; Mercer and Gregersen, 2025), findings from this study pointed to cultural immersion as a potentially distinctive environmental source of CFLE in AI-supported contexts.

Admittedly, although the interview sample included students from 6 countries, the small number of participants per nationality (ranging from 1 to 2) limited the scope for systematic cross-national comparison. While individual narratives suggested some variation in how students from different regions might engage with specific cultural content (e.g., Moroccan tea culture, Silk Road heritage), these informative observations were preliminary and could be further validated through investigation with larger, more balanced samples.

Overall, AI appeared to function as a supportive pedagogical medium rather than a direct source of enjoyment. CFLE may arise when AI support were perceived as facilitating immersive cultural experiences, social interaction, and learner agency in authentic cultural practices. This study tentatively extends current CFLE literature by indicating that CFLE appears to be more closely associated with meaningful cultural participation than with the novelty of AI support or superficial cultural exposure.

### 5.3 An SDT-informed interpretation of CFLE experience

As interpreted through a self- determination theory (SDT) lens, CFLE seemed to arise from the satisfaction of three psychological needs, with teachers being perceived acting as an essential mediator between AI-supported immersive resources and positive learning emotions, an inference drawn from participants’ accounts, since autonomy, competence, and relatedness were not directly measured. AI support and cultural immersion primarily served as sources of learning materials within the present context; on their own, they appeared to have limited capacity to satisfy learners’ relatedness, competence, or autonomy without instructional mediation.

Relatedness appeared to be the most salient factor shaping learners’ CFLE, perceived by participants to be largely nurtured through teacher practice. Responsive instructors were perceived to mediate by building trusting relationships and designing group activities and cultural field trips to foster a supportive community (Chiu et al., 2024; Ryan and Deci, 2020). This mediation of relational support was perceived vital for CFL learners adjusting to unfamiliar linguistic and cultural surroundings.

Learners’ sense of competence was perceived as closely associated with teacher mediation through scaffolding and positive feedback. While AI was perceived as supporting cultural practice, feelings of mastery broadly came from encouragement mediated by teachers. Unlike traditional L2 research that mainly focused on linguistic skills, this proposed model seeks to highlight the relatively underexplored cultural competence as a key source of fulfilment (Ryan and Deci, 2024).

Findings suggested that AI-supported flexible platforms alone may be insufficient to foster learner autonomy without additional human-mediated support. Instead, teachers appeared to mediate by unlocking autonomous exploration through assigning open-ended cultural tasks and permitting personalised inquiry within simulations, which aligned with scholarship suggesting connections between teacher-mediated autonomy and intrinsic motivation (Noels et al., 2019; Ryan and Deci, 2020; Wang, 2024).

Interpreted from participants’ accouts, teachers were seen to serve a mediating function through practices that may correspond to the satisfaction of the three SDT psychological needs: interpersonal care built relatedness, targeted feedback boosted competence, and guided AI exploration nurtured autonomy. Coordinated cultural activities were also perceived simultaneously to strengthen learners’ agency, confidence and belonging, aligning with prior SDT-related language learning research (Ryan and Deci, 2020; Noels et al., 2019).

The proposed teacher-mediated concept tentatively extends AI-supported SDT literature which presumes AI directly shapes learner motivation (Li et al., 2025; Chang and Sun, 2024). This study offers an alternative perspective on the assumption that psychological need satisfaction is instructionally mediated, not technologically determined: AI creates only potential learning opportunities; teachers are the critical mediators who connect digital tools, meaning construction, and enduring enjoyment. This claim receives partial corroboration yet requires qualification in Zhang et al. (2026), whose study identifies Enthusiastic Integrators as evidence that GenAI can foster positive affect. However, the coexistence of three less sanguine profiles, Task-Oriented Pragmatists, Conditional Adopters, and Self-Directed Writers, suggests that such positivity is episodic and profile-dependent, tethered to individual disposition rather than instructional design. The present finding advances this conversation by showing that from participants’ accounts, teacher mediation emerged as a potentially pivotal contributing variable, an SDT- informed interpretation: AI supports positive emotion in language learning not autonomously, but only when teachers actively mediate by scaffolding, regulating, and humanizing the GenAI-assisted writing process. In so doing, teacher mediation may transform what Zhang et al. (2026) treat as fixed learner typologies into pedagogically cultivable outcomes, thereby likely to shift the locus of affective agency from AI support to AI-supported instruction.

Accordingly, the findings may be synthesized into an interpretive framework for understanding the experience of CFLE through the lens of SDT (see Figure 1). From this perspective, participation in an AI-supported immersive Chinese culture course is likely to provide opportunities for authentic cultural engagement, while teacher mediation can transform these experiences into meaningful learning opportunities that can be interpreted as supporting learners’ needs for autonomy, competence, and relatedness. Viewed collectively, these interconnected processes may offer one plausible explanation for how CFLE may develop within this instructional context.

**Figure 1:**
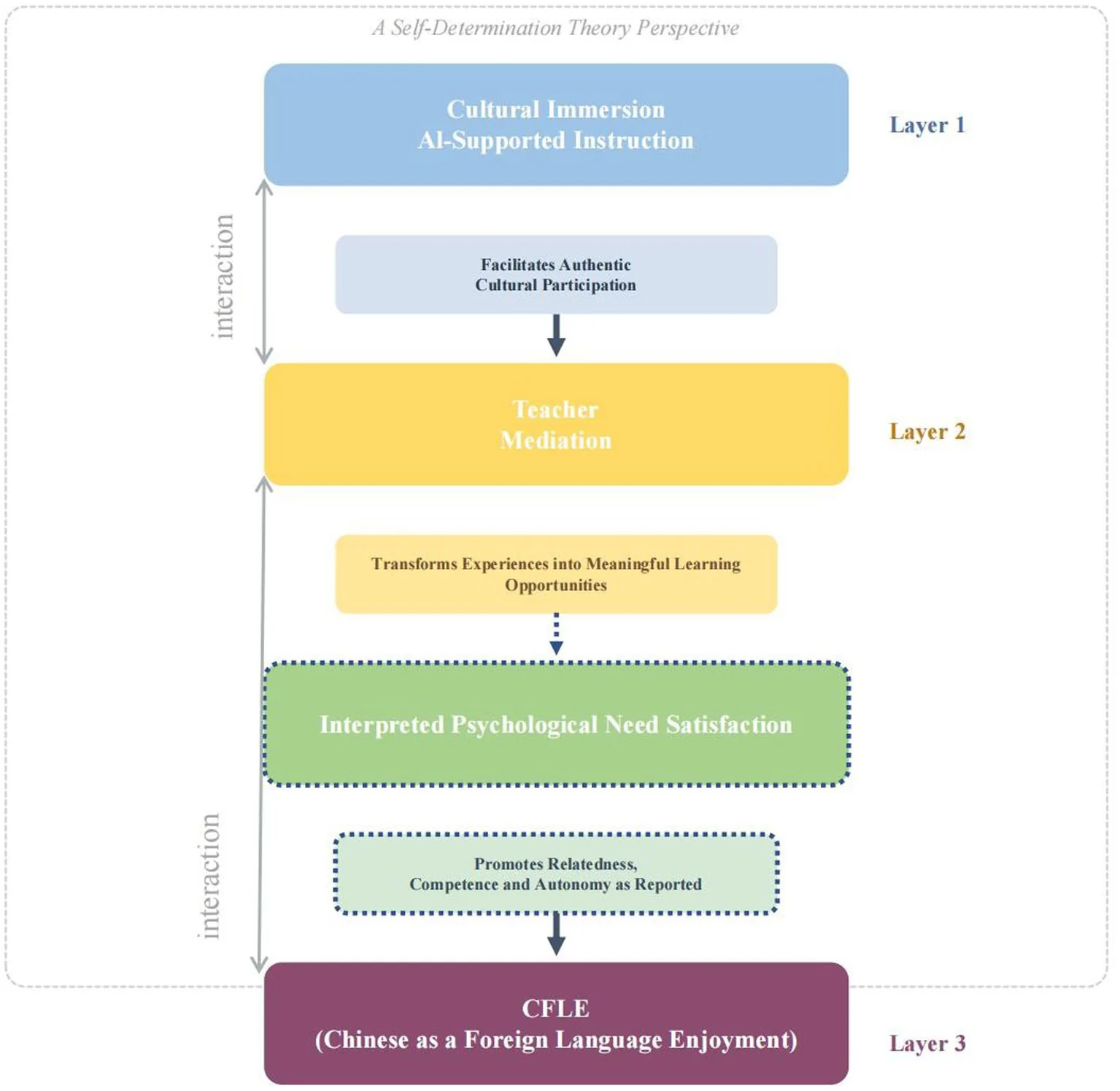
An interpretive framework illustrating teacher-mediated support for the CFLE experience from an SDT perspective. Psychological need satisfaction constitutes a theoretically inferred construct rather than an empirically measured layer.

As an interpretive framework rather than an empirically validated model, Figure 1 can be read as one possible theoretically informed account of the mechanisms that may underlie positive emotional experiences in AI-supported culturally immersive CFL learning. It further suggests that the value of AI may lie not merely in its technological novelty or capabilities but in its capacity to facilitate culturally authentic, socially meaningful, and psychologically supportive learning through effective teacher mediation.

## 6 Limitations and future research

While the present study achieved its intended aims, several limitations should be acknowledged and may serve as directions for future research. First, the study was conducted within a specific AI-supported immersive Chinese culture course that integrated language learning, cultural immersion, and technological support. As a result, it is difficult to isolate the unique contribution of individual instructional components. The positive emotional experiences observed may have resulted from AI technologies, cultural activities, teacher mediation, or their combined effects. Second, although the adapted questionnaire demonstrated high reliability and internal consistency, the questionnaire was employed primarily as a descriptive tool to contextualize participants’ overall positive emotions. Due to the explanatory nature of this study and the modest sample size (*N* = 82) relative to the number of items (*k* = 40), no inferential statistical analyses (e.g., factor analysis, or regression) but correlational analyses were conducted. Findings from the questionnaire should therefore be interpreted as descriptive only and are not used to support causal or comparative claims. Future research should disentangle these influences by comparing different levels of technological integration and cultural immersion and by refining measurement instruments to investigate causal or comparative claims. Third, whether FLE is best conceptualized as a unitary or differentiated construct remains a theoretical question. Current scholarship largely supports the latter view, proposing that FLE comprises distinguishable teacher-, learner-, and environment-associated components that can be measured empirically. However, the high correlations among categories in the data suggest that, at least in the present sample and context, learners may not clearly distinguish among these sources in their subjective experience. Enjoyment may be experienced holistically, with all three components contributing to a single affective state. Through our explanatory study, we tend to conclude that the present questionnaire does not strongly validate the three-component model as distinct psychological categories. Future research might pursue this question with alternative methods: experimental designs that isolate teacher versus environmental influences, qualitative interviews that probe learners’ causal attributions, or longitudinal designs that track whether different sources of enjoyment diverge over time. Furthermore, the study did not directly measure learners’ needs for autonomy, competence, and relatedness, although Self-Determination Theory (SDT) provided a useful framework for interpreting the findings. Consequently, the SDT-informed explanations remain inferential rather than empirically tested. Future studies should incorporate validated measures of psychological need satisfaction, and employ mediation or structural equation modeling to examine the proposed mechanisms more directly, directly measuring SDT need satisfaction and empirically testing the proposed relationships across diverse CFL learning contexts. In addition, the interview participants were selected from those who had volunteered to be interviewed, which may have introduced a positivity bias. As students with more negative experiences might have been less likely to volunteer, future studies should incorporate systematic strategies to capture negative cases. Finally, the sample consisted primarily of international students from Morocco, South Africa, Kazakhstan, Kyrgyzstan, Tajikistan, etc. While this population provides valuable insight into CFL learning in AI-supported culturally immersive environments, the findings may not be generalizable to learners from other linguistic, educational, and cultural backgrounds. Future research should examine CFLE across more diverse learner populations and instructional settings.

## 7 Conclusion

This study adopted a sequential explanatory mixed-methods design to explore Chinese Foreign Language Enjoyment (CFLE) among international students enrolled in an AI-supported immersive Chinese culture course. Drawing on the questionnaire and interview data, it examined CFLE structural patterns and learners’ affective experiences through the lens of Self-Determination Theory (SDT).

Results indicated moderately high CFLE levels across the AI-supported culturally immersive learning context, with teacher-related enjoyment as the highest-rated category, followed by learner- and environment-related enjoyment. Qualitative thematic patterns further tentatively bore witness to an association between learners’ enjoyment and supportive teacher mediation, teacher-student interactions, immersive cultural participation, and AI-based instructional support within the context of this study.

Importantly, AI was perceived to function as a complementary pedagogical tool rather than a teacher substitute. Although AI is capable of delivering instant feedback and individualized practice, teachers appeared to serve an important mediating function by providing emotional scaffolding, cultural mediation, and motivational regulation. These findings largely confirm that AI support alone is unlikely to sustain long- term CFLE; instead, CFLE tends to emerge from the interplay among interpersonal and AI resources support.

An SDT-informed lens suggested that differentiated patterns of psychological need satisfaction may be associated with CFLE. In AI- supported culturally immersive settings, relatedness was perceived to be most salient through teacher - student and peer connections, while competence was at times vulnerable to ambiguous AI - generated feedback; autonomy satisfaction was perceived to be heavily shaped by teacher mediation.

Accordingly, this study attempts an alternative conceptualization of AI-supported culturally immersive CFLE as a teacher-mediated, learning ecosystem integrating instructional design, sociocultural engagement, and SDT-informed need satisfaction (see Figure 1). This framework offers a provisional illustration of multi-source CFLE experience through balanced autonomy, competence, and relatedness fulfilment within cultural learning contexts. Theoretically, this work seeks to extend CFLE research by complementing quantitative measurement with a teacher-mediated ecosystemic lens, inviting a reconsideration of the assumption that AI can independently sustain learner motivation. Practically, it yields tentative, evidence-based strategies to inform the design of culturally grounded, AI-supported immersive environments conducive to positive emotional-motivational experiences among CFL learners.

## Data Availability

The original contributions presented in the study are included in the article/supplementary material, further inquiries can be directed to the corresponding authors.
